# Diagnosis of non-occlusive acute mesenteric ischemia in the intensive care unit

**DOI:** 10.1186/s13613-016-0213-x

**Published:** 2016-11-17

**Authors:** Simon Bourcier, Ammar Oudjit, Geoffrey Goudard, Julien Charpentier, Sarah Leblanc, Romain Coriat, Hervé Gouya, Bertrand Dousset, Jean-Paul Mira, Frédéric Pène

**Affiliations:** 1 Service de Réanimation Médicale, Hôpital Cochin, Hôpitaux Universitaires Paris Centre, Assistance Publique-Hôpitaux de Paris, 27 rue du Faubourg Saint-Jacques, 75014 Paris, France; 2 Service de Radiologie A, Hôpital Cochin, Hôpitaux Universitaires Paris Centre, Assistance Publique-Hôpitaux de Paris, Paris, France; 3 Service de Chirurgie Digestive, Hôpital Cochin, Hôpitaux Universitaires Paris Centre, Assistance Publique-Hôpitaux de Paris, Paris, France; 4 Service de Gastro-entérologie, Hôpital Cochin, Hôpitaux Universitaires Paris Centre, Assistance Publique-Hôpitaux de Paris, Paris, France; 5 Faculté de Médecine, Université Paris Descartes, Paris, France

**Keywords:** Intensive care unit, Ischemia, Mesenteric, Surgery, Endoscopy, CT-scan

## Abstract

**Background:**

Non-occlusive mesenteric ischemia (NOMI) is a common complication and accounts for a major cause of death in critically ill patients. The diagnosis of NOMI with respect to the eventual indications for surgical treatment is challenging. We addressed the performance of the diagnostic strategy of NOMI in the intensive care unit, with emphasis on contrast-enhanced abdominal CT-scan.

**Methods:**

This was a retrospective monocenter study. Patients with clinically suspected acute mesenteric ischemia were included if a comprehensive diagnostic workup was carried out including surgical and/or endoscopic digestive explorations. Patients with evidence of occlusive mesenteric ischemia were excluded. A definite diagnosis of NOMI only relied on surgical or endoscopic findings. Abdominal CT-scans were reviewed by two radiologists blinded from the final diagnosis.

**Results:**

A diagnosis of NOMI could be definitely confirmed or ruled out through surgical or endoscopic explorations of the digestive tract in 147 patients. With respect to their clinical characteristics, only a history of atrial fibrillation was an independent predictor of NOMI (odds ratio 8.3, 95% confidence interval 2.0–35.2, *p* = 0.004). Among them, 114 patients (75 with and 39 without NOMI) had previously been subjected to contrast-enhanced abdominal CT-scan. Portal venous gas, pneumatosis intestinalis and, to a lesser extent, abnormal contrast-induced bowel wall enhancement were poorly sensitive, but exhibited good specificities of 95, 85 and 71%, respectively. Nineteen out of 75 patients (25.3%) without any suggestive radiological signs finally exhibited mesenteric ischemia, including ten with intestinal necrosis.

**Conclusions:**

The performance of abdominal CT-scan for the diagnosis of NOMI is limited. Radiological signs of advanced-stage ischemia are good predictors of definite mesenteric ischemia, while their absence should not be considered sufficient to rule out the diagnosis.

**Electronic supplementary material:**

The online version of this article (doi:10.1186/s13613-016-0213-x) contains supplementary material, which is available to authorized users.

## Background

Acute mesenteric ischemia (AMI) is a dreaded complication in critically ill patients and remains a major diagnostic and therapeutic challenge in most cases. Importantly, AMI encompasses two different pathophysiological entities. Occlusive AMI is caused by the occlusion of large mesenteric arteries or veins due to arterial embolism or a local thrombotic process. AMI may also occur despite preserved patency of large mesenteric vessels, the so-called non-occlusive mesenteric ischemia (NOMI) [1]. NOMI is a common complication in critically ill patients with acute circulatory failure and thereby accounts for a major cause of death in the intensive care unit (ICU) [2, 3]. In a large multicenter study of 780 ICU patients with AMI, the overall mortality rate was 58% [4]. Nonetheless, surgical treatment within 24 h of diagnosis of AMI was identified as an independent predictor of survival, emphasizing the importance of early and reliable diagnosis.

The diagnosis and the treatment of AMI rely on a timely multidisciplinary management involving intensive care physicians, gastroenterologists, radiologists and surgeons [5]. The diagnosis of AMI is often challenging in critically ill patients, most especially for NOMI. It can be suspected in the presence of clinical deterioration associated with digestive symptoms and biological manifestations suggestive of profound tissue ischemia or acute cell lysis. Contrast-enhanced abdominal CT-scan is the cornerstone of the diagnostic strategy and may provide direct or indirect arguments for impaired vascularization of the bowel [6]. However, its accuracy for the diagnosis of NOMI in critically ill patients is questionable. A confirmatory diagnosis as well as the assessment of the extent of necrosis still commonly involves a direct visualization of the digestive tract by endoscopy and/or surgical exploration.

With respect to the frequent diagnostic uncertainty of NOMI in critically ill patients, a better assessment of the preoperative probability of mesenteric ischemia as well as the eventual possibilities of surgical treatment represents an important area of improvement in the management of the disorder. To this aim, we herein addressed the performance of the common diagnostic strategy of NOMI in the ICU, with particular emphasis on abdominal CT-scan.

## Patients and methods

### Patients and setting

We performed a retrospective monocenter study over an 8-year period (2007–2013) in a 24-bed tertiary medical ICU. The average number of admissions is 1600 per year, and the case-mix is distributed into 90% of medical patients and 10% of patients requiring emergency surgery at the time of admission or during the stay in the ICU. Patients with clinically suspected AMI were included if a comprehensive diagnostic workup including surgical and/or endoscopic explorations of the digestive tract was carried out, regardless of previous abdominal CT-scan imaging. Patients with evidence of occlusive AMI (i.e., interrupted blood flow in large mesenteric vessels) were excluded. This study was part of a project approved by the ethics committee of the French Intensive Care Society. Informed consent was waived due to the retrospective observational design of the study.

### Intended management of AMI

In our unit, the diagnosis of AMI relies on a multidisciplinary approach involving intensive care physicians, gastroenterologists, radiologists and surgeons. AMI was commonly suspected on the basis of clinical upper or lower digestive symptoms including abdominal pain, feeding intolerance, gastrointestinal hemorrhage, diarrhea, occlusion, associated or not with deterioration of organ failures, and biological manifestations of tissue ischemia (elevated arterial lactate levels) and cell lysis (increased serum levels of lactate dehydrogenase, creatine phosphokinase and transaminases). A moderate to high probability for AMI prompted further digestive investigations by contrast-enhanced abdominal tomodensitometry and/or upper and lower endoscopic explorations and/or laparotomy. Intestinal resection was indicated in case of localized bowel necrosis. The major steps of patients’ management such as indications for major surgery, transfer to the operating room, or decisions of withholding or withdrawing life support were discussed collectively.

### Diagnostic criteria of acute mesenteric ischemia

**Fig. 1:**
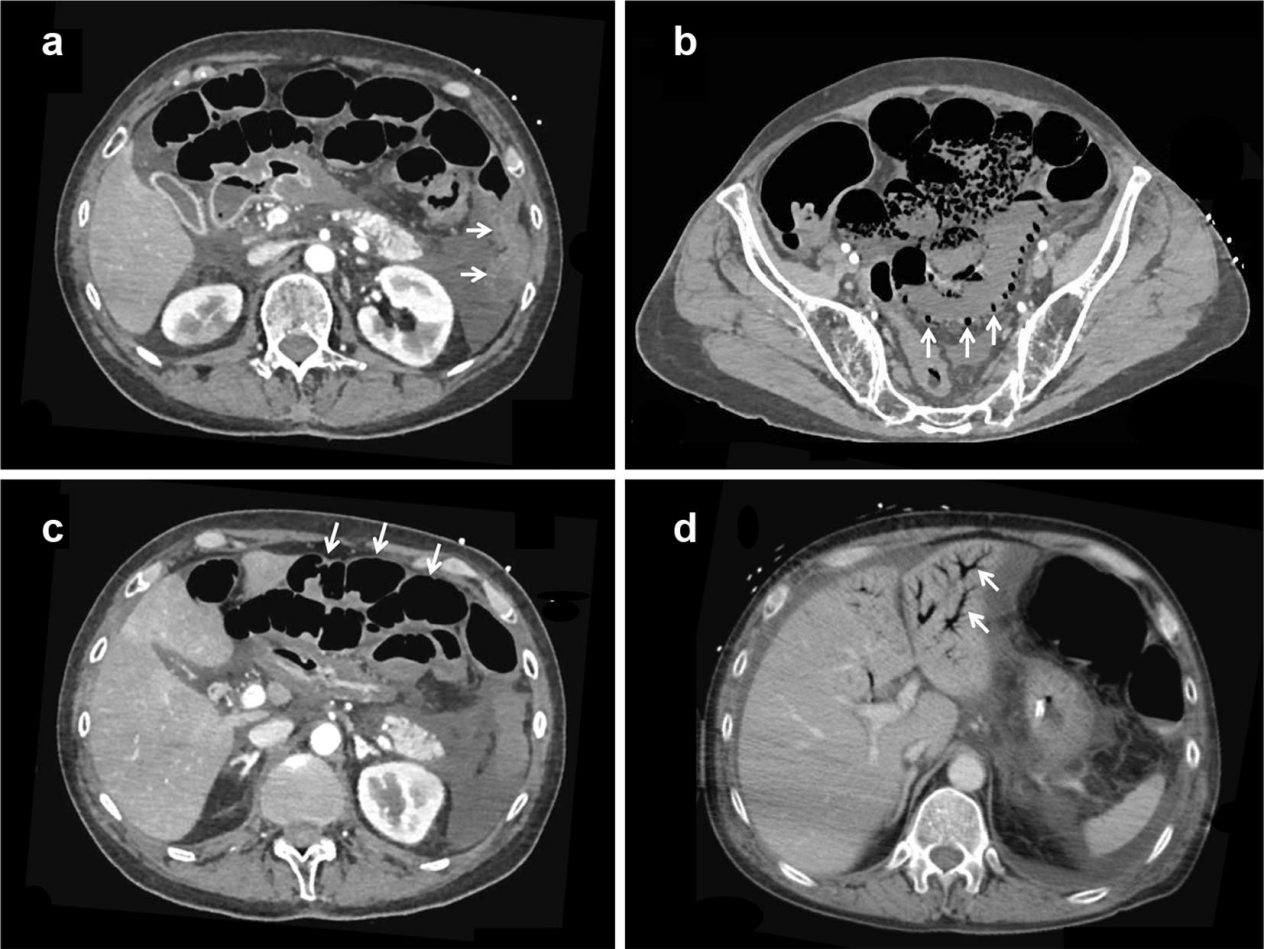
Representative CT-scan findings of non-occlusive mesenteric ischemia. **a** Absence of contrast-induced bowel wall enhancement (*arrows*). **b** Pneumatosis intestinalis and absence of contrast-induced bowel wall enhancement (*arrows*). **c** Bowel dilatation and absence of contrast-induced bowel wall enhancement (*arrows*). **d** Portal venous gas (*arrows*)

Two radiologists (AO and HG) blinded from the final diagnosis reviewed all abdominal CT-scans. Multidetector CT-scan were first carried out without contrast and secondarily contrast-enhanced with early (arterial time 30 s) and delayed (portal venous time 60–70 s) acquisitions following intravenous contrast medium infusion. The following radiological signs were systematically collected: pneumatosis intestinalis defined by the presence of gas inside the bowel’s walls, bowel dilatation, portal venous gas, aortic or mesenteric atherosclerosis, lack or heterogeneity of contrast-induced enhancement of bowel’s walls (Fig. 1). All eventual operative and endoscopic report forms were reviewed by investigators (GG and SL) blinded from the CT-scan findings. Regardless of CT-scan, only undisputed mesenteric ischemia diagnosed by surgical or endoscopic explorations were classified as definite NOMI. Extensive ischemia was defined as digestive ischemia involving more than one digestive segment. Conversely, NOMI was ruled out when neither surgical nor endoscopic explorations retrieved macroscopic evidence of digestive ischemia.

### Collection of data

The following data were collected, either extracted from our patient’s data management system (Centricity CliniSoft, GE Healthcare) or retrieved from individual medical files: demographics (age and gender), underlying comorbidities including cardiovascular diseases, the primary diagnosis warranting ICU admission, the severity of illness at the time of ICU admission as assessed by the Simplified Acute Physiology Score II (SAPS II) and the Sequential Organ Failure Assessment (SOFA) scores [7, 8]. Features associated with clinically suspected NOMI were the following: time from admission to clinical suspicion (i.e., to the first digestive exploration, CT-scan, surgery or endoscopy), digestive symptoms, arterial blood lactate, serum enzymes levels (lactate dehydrogenase, creatine phosphokinase, transaminases), concurrent organ failures as quantified by the SOFA score.

### Statistical analysis

Statistical analyses were performed using the software Prism 5.0 (Graphpad, San Diego, CA). Categorical data are presented as numbers (%) and compared by Chi-square or Fisher’s exact test as appropriate. Continuous variables are expressed as median and interquartile range and compared using the nonparametric Mann–Whitney test. Variables found associated with a *p* value <0.20 in univariate analysis were entered into a multivariate backward stepwise logistic regression analysis in order to identify the factors independently associated with a definite diagnosis of NOMI. The goodness-of-fit of the model was checked by the Hosmer–Lemeshow test.

## Results

**Fig. 2:**
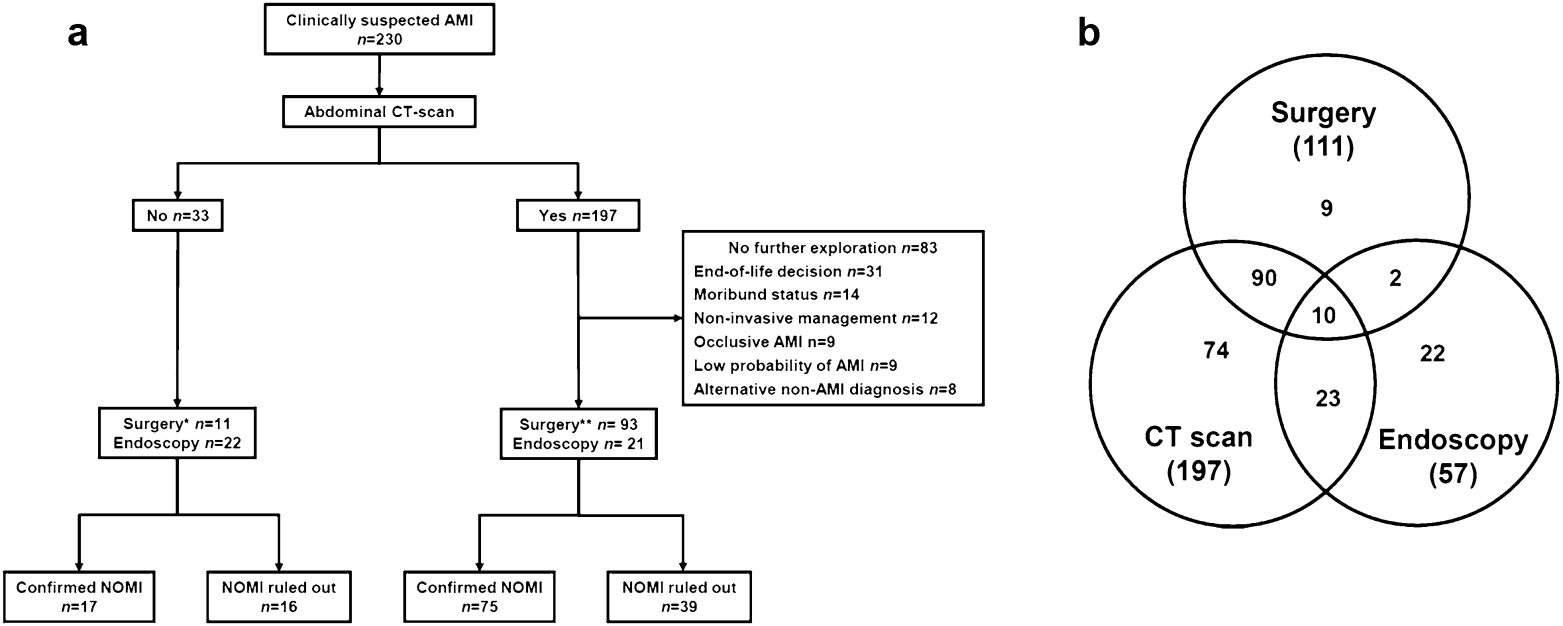
Investigations for acute mesenteric ischemia. **a** Flowchart of the study. Including 2 (*) and 10 (**) patients for whom both laparotomy and endoscopy were performed. **b** Distribution of diagnostic procedures. *AMI* acute mesenteric ischemia, *NOMI* non-occlusive mesenteric ischemia

AMI was suspected in 230 patients on the basis of clinical and biological manifestations and prompted some specific digestive investigations. Most patients (197/230, 85%) were explored by contrast-enhanced abdominal CT-scan and were secondarily subjected to further digestive explorations by surgery (*n* = 93 including 10 patients who also had digestive endoscopy) or by endoscopy only (*n* = 21) (Fig. 2). Of note, nine patients were excluded because the CT-scan displayed evidence of occlusive AMI with interrupted blood flow within the upper mesenteric artery. The characteristics of the 83 patients who had CT-scan without further exploration are shown in the Additional file 1: Table S1. Thirty-three patients did not have CT-scan and were directly investigated by surgery (*n* = 11 including two patients who also had digestive endoscopy) or by endoscopy only (*n* = 22). Altogether, surgery and digestive endoscopy led to a definite diagnosis of NOMI in 92 patients (70 and 22 patients, respectively) and ruled it out in 55 patients.

The characteristics of patients with and without NOMI are displayed in Table 1. NOMI was mostly ICU-acquired since diagnosed at 3.9 (1.5–12.9) days from ICU admission. Patients with definite NOMI were older and were less likely to have diabetes and more likely to have atrial fibrillation. The primary causes for ICU admission as well as the initial severity of illness were similar. Clinical symptoms and biological manifestations hardly discriminated the patients with definite NOMI from those for whom NOMI could be ruled out. In a multivariate logistic regression analysis adjusted with the other factors (age, gender, diabetes, body mass index, creatine phosphokinase level, concurrent antibiotic treatment) that reached a *p* value <0.2 in the univariate analysis (Table 1), only a history of atrial fibrillation was an independent predictor of NOMI (odds ratio 8.3, 95% confidence interval 2.0–35.2, *p* = 0.004). The Hosmer–Lemeshow goodness-of-fit assessment of the final model reported a *p* value of 0.90.

**Table 1:** Characteristics of patients with and without non-occlusive mesenteric ischemia

Characteristics	Definite NOMI (*n* = 92)	NOMI ruled out (*n* = 55)	*p*
Baseline characteristics			
Age (years)	75 (61–81)	66 (55–78)	0.02
Male gender	41 (45%)	31 (56%)	0.18
BMI (kg/m^2^)	24 (21–27)	26 (23–29)	0.15
Comorbidities			
Diabetes	13 (14%)	16 (29%)	0.03
Hypertension	50 (54%)	27 (49%)	0.61
Smoking	43 (47%)	26 (47%)	1.0
Coronary disease	26 (28%)	20 (36%)	0.36
Peripheral vascular disease	14 (15%)	12 (22%)	0.37
End-stage renal disease	7 (8%)	4 (7%)	1.0
Atrial fibrillation	33 (36%)	12 (22%)	0.10
Main diagnosis at ICU admission			0.90
Severe sepsis or septic shock	41 (45%)	22 (40%)	
Cardiogenic shock	3 (3%)	4 (7%)	
Hypovolemic shock	14 (15%)	7 (13%)	
Hemorrhagic shock	9 (10%)	7 (13%)	
Acute kidney injury	3 (3%)	3 (5%)	
Cardiac arrest	13 (14%)	7 (13%)	
Cardiac surgery	9 (10%)	5 (9%)	
Illness severity at admission (points)			
SAPS II	70 (57–87)	74 (48–91)	0.75
SOFA	8 (5–11)	8 (4–12)	0.78
Diagnosis of NOMI			
Time to diagnosis (days)^a^	3.9 (1.5–13.0)	4.7 (2.1–9.7)	0.68
Concurrent anticoagulation			0.75
Preventive	34 (37%)	17 (31%)	
Curative	19 (20.7%)	13 (23.6%)	
Concurrent antibiotic treatment	90 (97.8%)	46 (83.6%)	0.002
SOFA (points)	10 (6–13)	9 (7–12)	0.97
Clinical manifestations			
Lower digestive symptoms	56 (61%)	29 (53%)	0.39
Upper digestive symptoms	38 (41%)	19 (35%)	0.49
Bacteremia	20 (21.7%)	9 (16.4%)	0.43
Serum laboratory results			
Bicarbonates (mmol/L)	16.0 (14.0–21.1)	17.0 (14.5–21.5)	0.83
Arterial lactate (mmol/L)	5.6 (2.2–10.1)	6.3 (2.1–11.2)	0.60
Creatinine (μmol/L)	159 (125–231)	132 (84–247)	0.29
K^+^ (mmol/L)	4.6 (4.1–5.4)	4.5 (3.9–5.2)	0.46
CPK, ×UNV	1.0 (0.4–5.6)	3.0 (7.8–9.1)	0.09
LDH, ×UNV	4.9 (2.2–12.7)	7.5 (2.8–17.3)	0.35
AST, ×UNV	7.8 (1.3–43.7)	3.0 (1.0–43.5)	0.30
Leukocyte count (G/L)	14.1 (9.5–23.6)	15.3 (8–22.5)	0.96
Hemoglobin (g/dL)	10.0 (8.9–11.3)	9.9 (8.9–11.2)	0.51
Platelets (G/L)	120 (61–199)	117 (62–182)	0.94
ICU mortality	69 (76%)	27 (49%)	0.002

Categorical variables are expressed as median (interquartile range)

Lower digestive symptoms include hematochezia, melena and diarrhea. Upper digestive symptoms include vomiting, feeding intolerance and acute upper gastrointestinal bleeding

*BMI* body mass index, *NOMI* non-occlusive mesenteric ischemia, *ICU* intensive care unit, *SAPS II* simplified acute physiology score II, *SOFA* sequential organ failure assessment, *UNV* upper normal value (IU/L)

^a^Time from ICU admission to the first investigation of NOMI (CT-scan, surgery or endoscopy)

Intestinal necrosis was the main macroscopic aspect observed during laparotomy (77.1% of patients) (Table 2). Ischemia could be located to every intestinal segment and involved more than one segment in 37.1% of cases. A surgical treatment by segmental intestinal resection was possible for 47 patients (67.5% of operated patients). A definite diagnosis of NOMI was associated with a poor prognosis with an in-ICU mortality rate of 76%.

**Table 2:** Diagnostic features of non-occlusive mesenteric ischemia

	Number of patients (%)
Location	92 (100)
Stomach and/or duodenum	30 (32.6)
Jejunum and/or ileum	44 (47.8)
Right colon	50 (54.3)
Left colon	46 (50)
Sigmoid colon	39 (42.4)
Rectum	12 (13)
Surgical findings	70 (100)
Peritonitis	15 (21.4)
Perforation	14 (20)
Peritoneal effusion	48 (68.6)
Necrosis	54 (77.1)
Non-necrotic ischemic lesions	16 (22.9)
Extent of ischemia	
Extensive (≥2 intestinal segments)	28 (40)
Segmental	42 (60)
Digestive endoscopy	29 (100)
Upper digestive fibroscopy	12 (41.4)
Colonoscopy	12 (41.4)
Rectosigmoidoscopy	5 (17.2)
Endoscopic findings	29 (100)
Ischemia	19 (65.5)
Necrosis	10 (34.5)

With respect to the challenging diagnosis of NOMI in this setting, we addressed the diagnostic performance of abdominal CT-scan in the 114 patients who were subjected to both contrast-enhanced abdominal CT-scan and one of the reference investigations, either surgery or digestive endoscopy (Fig. 2). Among them, NOMI was definitely confirmed or ruled out in 75 and 39 patients, respectively. The individual assessments of radiological signs are presented in Table 3 and in the Additional file 1: Table S2. Aortic or mesenteric atherosclerosis was present in most patients and thereby had very good sensitivity, but poor specificity. Portal venous gas, pneumatosis intestinalis and, to a lesser extent, abnormal contrast-induced bowel wall enhancement were poorly sensitive, but exhibited good specificities of 95, 85 and 71%, respectively. These three radiological signs were often combined in patients with NOMI (Additional file 1: Figure S1). Of note, 19 out of 75 patients (25.3%) without any suggestive radiological signs finally exhibited mesenteric ischemia (13 diagnosed by surgery and 6 by endoscopy), resulting in intestinal necrosis in ten patients.

**Table 3:** Performance of abdominal CT-scan findings in the diagnosis of non-occlusive mesenteric ischemia

Radiological sign	Definite NOMI (*n* = 75) (%)	NOMI ruled out (*n* = 39) (%)	Odds ratio	*p*	Sensitivity %	Specificity %	PPV %	NPV %
Abnormal wall enhancement	62.0	28.6	4.07 (1.70–9.78)	<0.001	62 (50–73)	71 (54–85)	81 (69–91)	48 (34–62)
Pneumatosis intestinalis	32.4	15.4	2.64 (0.97–7.16)	0.07	32 (22–44)	85 (69–94)	80 (61–92)	40 (29–51)
Bowel dilatation	62.2	56.4	1.27 (0.58–2.79)	0.69	62 (50–73)	44 (28–60)	68 (55–78)	38 (24–53)
Portal venous gas	18.9	5.1	4.32 (0.93–20.09)	0.05	19 (11–30)	95 (83–99)	88 (62–98)	38 (28–49)
Atherosclerosis	90.6	82.0	2.09 (0.68–6.48)	0.23	91 (81–96)	18 (8–34)	68 (58–77)	50 (23–77)

Values in brackets represent the 95% confidence interval

*NOMI* non-occlusive mesenteric ischemia, *NPV* negative predictive value, *PPV* positive predictive value

## Discussion

AMI encompasses two different pathophysiological entities affected with distinct diagnostic and therapeutic issues. The diagnosis of occlusive AMI is often obvious, relying on the blockage of blood flow within large mesenteric arteries or veins on a contrast-enhanced CT-scan. The treatment combines surgical or instrumental arterial reperfusion and the eventual resection of necrotic bowel segments [9, 10]. In contrast, NOMI occurs despite seemingly preserved mesenteric blood flow and is most often associated with advanced age, cardiac insufficiency, atrial fibrillation, hemodialysis and prior episodes of hypotension [11–14]. It represents a significant complication in critically ill patients with severe and prolonged acute circulatory failure [2, 3]. However, prolonged hypoperfusion does not recapitulate the mechanisms that contribute to the advent of NOMI, that also involves mesenteric vasoconstriction, intestinal hypoxia while increased intestinal metabolic demand, ischemia–reperfusion injury, apoptosis and decreased proliferation of enterocytes [15]. Tissue necrosis and disruption of the intestinal barrier may then result in bacterial translocation, systemic inflammatory response and multiple organ failure [11].

Regardless of the underlying ischemic process, occlusive or non-occlusive, the prognosis of AMI is poor as the overall mortality rate may reach 80% in most published cohorts [3, 16–18]. Leone and colleagues provided reliable prognostic data for AMI in an impressive multicenter series of 780 cases [4]. The in-ICU mortality rate was 58%, and the possibility of an initial surgical treatment was identified as an independent predictor of survival. However, an important limit of the paper lies in the absence of formal diagnostic criteria for AMI. Indeed, the diagnosis of AMI could be supported by gastrointestinal endoscopy or surgery, or alternatively by CT-scan only in the majority (58%) of patients. Furthermore, the mechanism of mesenteric ischemia, occlusive versus non-occlusive, was not reported either.

Despite a better awareness of the disorder, the diagnosis of NOMI remains particularly challenging in critically ill patients, and diagnostic uncertainty may ultimately require surgical explorations for an accurate assessment of the bowel. Every effort should be made to refine the diagnostic performance for AMI, in order to avoid unnecessary and aggressive surgical interventions in patients without digestive ischemia, and also in patients with end-stage extensive necrosis ineligible for segmental intestinal resection. We herein assessed a stringent diagnostic workup in which AMI could only be definitely confirmed or ruled out by direct visualization of the digestive tract by endoscopic or surgical explorations. We addressed the performance of the main diagnostic steps including clinical and biological manifestations and abdominal CT-scan. Clinical symptoms and biological markers of tissue ischemia and acute cell lysis may commonly suggest the diagnosis of mesenteric ischemia, but clearly lack sensitivity and specificity in this setting [19, 20]. More relevant information is expected from abdominal CT-scan [6].

The performance of CT-scan has been reported as good to excellent in the diagnosis of AMI with sensitivity and specificity, generally about 90% [6]. However, it should be emphasized that these studies were performed in the setting of occlusive AMI, in which the diagnosis is made much easier by the blockade of mesenteric blood flow. In contrast, few studies have addressed its diagnostic value in NOMI. We report here that abdominal CT-scan has limited performance in this setting. Portal venous gas or pneumatosis intestinalis appeared as specific radiological signs (specificities 95 and 85%, respectively). Nonetheless, both may be seen in non-ischemic conditions including connective tissue disorders, inflammatory bowel diseases, cytotoxic chemotherapy and major bowel distension [21]. Abnormal wall enhancement was associated with a lower but still reasonable specificity of 71%. However, a major limit of those three CT-scan signs lied in their poor sensitivity (19, 32 and 62%, respectively), making their diagnostic contribution irrelevant in most patients.

Which clinical implications can be drawn from our findings? Although we pointed out the limitations of CT-scan, we keep thinking that it should remain the cornerstone of the diagnostic workup of AMI in the ICU. However, only a surgical exploration is currently able to provide an accurate assessment of bowel viability and to delineate the frankly necrotic sections requiring segmental intestinal resection. On the other hand, partially ischemic bowel regions might be liable to salvage after intraoperative visualization of restored mesenteric perfusion, spontaneously or after injection of a fluorescent dye [22]. The presence of portal venous gas or pneumatosis intestinalis surely indicates urgent exploratory laparotomy. The same should probably apply to abnormal wall enhancement. Finally, the still unanswered question remains about surgical indications in patients with negative abdominal imaging. Although it is difficult to provide firm recommendations, we would then propose a pragmatic approach in which surgical indications should be based on eventual clinical deterioration despite optimal medical treatment and advanced organ failure supports.

Finally, the most important treatment for NOMI is preventive and every effort should be made to limit the duration and severity of acute circulatory failure. Our therapeutic goals in this setting still remain largely based on macroscopic circulatory parameters, whereas microcirculatory disorders may also contribute to organ failures in an independent manner [23]. Reliable assessment of microcirculatory tissue perfusion in critically ill patients has been made possible by technological advances. However, the therapeutic implications remain questionable [24].

Our study has strengths and limitations that deserve to be mentioned. Its main strength lies in its stringent methodology despite its retrospective design. A definite diagnosis or conversely elimination of mesenteric ischemia required confirmatory investigations by surgery and/or endoscopy and could only be presumed on CT-scan. Furthermore, the investigators who interpreted the CT-scan imaging and those who confirmed the diagnosis of mesenteric ischemia through endoscopic and surgical reports were different and kept blinded from other concurrent investigations. With respect to limitations, this was a single-center study performed in a medical ICU setting where the large majority of patients are referred for a wide range of medical conditions. The retrospective design of the study may have led to incomplete identification of patients with suspected mesenteric ischemia. This is the reason why we only included patients with a significant clinical probability of AMI who were therefore explored by an abdominal CT-scan, or directly by endoscopy or surgery. In addition, urgent endoscopy can only explore the upper (oesophagus, stomach and duodenum) and lower (rectum and colon) extremities of the digestive tract, leaving unexplored the jejunum and ileum in between. We cannot exclude that some patients only explored by endoscopy without further surgery may still display mesenteric ischemia in non-visualized intestinal regions.

## Conclusions

AMI remains a diagnostic and therapeutic challenge in critically ill patients, especially in case of non-occlusive forms. The diagnostic contribution of abdominal CT-scan is limited in this setting. Radiological signs of advanced-stage ischemia represent undisputed indications for surgical intervention to assess the extent of bowel necrosis and the possibility of intestinal resection. The absence of radiological signs suggesting mesenteric ischemia should not be considered sufficient to rule out the diagnosis and still warrants further digestive explorations by endoscopy and/or laparotomy in case of high clinical suspicion.

## Additional file

**Additional file 1: Figure S1.** Distribution of radiological signs in patients with definite non-occlusive mesenteric ischemia. The Venn’s diagram was drawn from the 48 patients with definite non-occlusive mesenteric ischemia displaying at least one of the three most specific radiological signs. **Table S1.** Characteristics of patients who had CT-scan without further explorations. **Table S2.** Determinants of abnormal radiological signs in patients with definite non-occlusive mesenteric ischemia (n = 75).
