# Supplementary material for: Diagnosis of non-occlusive acute mesenteric ischemia in the intensive care unit
Source: Ann Intensive Care. 2016 Nov 17;6:112. doi: 10.1186/s13613-016-0213-x (PMC5114213; doi:10.1186/s13613-016-0213-x)
Supplement: Supplementary file 1 — Additional file 1: Figure S1. Distribution of radiological signs in patients with definite non-occlusive mesenteric ischemia. The Venn’s diagram was drawn from the 48 patients with definite non-occlusive mesenteric ischemia displaying at least one of the three most specific radiological signs. Table S1. Characteristics of patients who had CT-scan without further explorations. Table S2. Determinants of abnormal radiological signs in patients with definite non-occlusive mesenteric ischemia (n = 75). [file 13613_2016_213_MOESM1_ESM.docx]

**Diagnosis of non-occlusive acute mesenteric ischemia**

**in the intensive care unit**

Simon Bourcier, Ammar Oudjit, Geoffrey Goudard, Julien Charpentier,

Sarah Leblanc, Romain Coriat, Hervé Gouya, Bertrand Dousset,

Jean-Paul Mira, Frédéric Pène

**Table S1. Characteristics of patients who had CT-scan without further exporations**

**Table S2. Determinants of abnormal radiological signs in patients with definite non-occlusive mesenteric ischemia (n=75)**

**Figure S1. Distribution of radiological signs in patients with definite non-occlusive mesenteric ischemia**

**Table S1: Characteristics of patients who had CT-scan without further exporations**

| **Characteristics** | **No further exploration (n=83)** |
| --- | --- |
| **Baseline characteristics**  Age, years  Male gender  BMI, kg/m^2^  Comorbidities  Diabetes  Hypertension  Smoking  Coronary disease  Peripheral vascular disease  End-stage renal Disease  Atrial fibrillation  Main diagnosis at ICU admission  Severe sepsis or septic shock  Cardiogenic shock  Hypovolemic shock  Hemorragic shock  Acute kidney injury  Cardiac arrest  Cardiac surgery  Illness severity at admission, points  SAPS II  SOFA | 75 (62–82)  46 (55%)  25 (22–27)  16 (19%)  50 (60%)  39 (47%)  20 (24%)  10 (12%)  4 (5%)  38 (46%)  49 (59%)  4 (5%)  7 (8%)  3 (4%)  4 (5%)  8 (10%)  7 (8%)  70 (48–87)  8 (4–12) |
| **Suspected diagnosis of AMI**  Time to diagnosis, days ^a^  SOFA, points  Clinical manifestations  Lower digestive symptoms  Upper digestive symptoms  Serum laboratory results  Bicarbonates, mmol/L  Arterial lactate, mmol/L  Creatinin, μmol/L  K^+^, mmol/L  CPK, × UNV  LDH, × UNV  AST, × UNV  Leukocyte count, G/L  Hemoglobin, g/dL  Platelets, G/L | 1.9 (1.1–4.8)  9 (4–13)  34 (41%)  26 (31%)  18.6 (13.8–22.3)  4.4 (1.7–8.1)  128 (73–220)  4.1 (3.6–4.7)  1.4 (0.5–9.8)  3.8 (2.3–12.0)  3.2 (1.1–19.4)  11.3 (5.8–21.3)  10.4 (9.2–11.9)  105 (63–225) |
| **ICU Mortality** | 59 (71%) |

Abbreviations: BMI, body mass index; ICU Intensive Care Unit; SAPS II, simplified acute physiology score II; SOFA, sequential organ failure assessment; UNV, upper normal value (IU/L).

Lower digestive symptoms include hematochezia, melena and diarrhea. Upper digestive symptoms include vomiting, feeding intolerance and acute upper gastrointestinal bleeding.

^a^ Time from ICU admission to CT-scan

| Variables | Abnormal wall enhancement | |  | Pneumatosis intestinalis | |  | Portal venous gas | |  |
| --- | --- | --- | --- | --- | --- | --- | --- | --- | --- |
|  | **Yes (n=45)** | **No (n=30)** | ***p*** | **Yes (n=23)** | **No (n=52)** | ***p*** | **Yes (n=13)** | **No (n=62)** | ***p*** |
| Atrial fibrillation, n (%) | 15 (33.3%) | 11 (36.7%) | 0.61 | 10 (43.5%) | 18 (34.6%) | 0.80 | 6 (46.2%) | 22 (35.5%) | 0.75 |
| Arterial lactate level, mmol/L | 5.6 (2.6–10.5) | 7.3 (1.9–9.0) | 0.98 | 6.7 (2.4–11.3) | 4.9 (2.3–9.0) | 0.77 | 7.4 (5.8–10.8) | 4.3 (2.1–9.0) | 0.12 |
| Positive blood culture, n (%) | 13 (28.9%) | 3 (10%) | 0.09 | 6 (26.1%) | 11 (21.2%) | 0.77 | 4 (30.8%) | 13 (21.0%) | 0.50 |
| Anatomic location |  |  |  |  |  |  |  |  |  |
| Stomach / duodenum, n (%) | 13 (28.9%) | 14 (46.7%) | 0.03 | 8 (34.8%) | 13 (25%) | 0.78 | 5 (38.4%) | 16 (25.8%) | 0.74 |
| Jejunum / ileum, n (%) | 25 (55.5%) | 17 (56.7%) | 0.41 | 16 (69.6%) | 24 (46.2%) | 0.30 | 9 (69.2%) | 31 (50%) | 0.54 |
| Right colon, n (%) | 29 (64.4%) | 13 (43.3%) | 0.55 | 16 (69.6%) | 28 (53.8%) | 0.77 | 9 (69.2%) | 35 (56.5%) | 1.0 |
| Left colon / rectum, n(%) | 26 (57.8%) | 16 (53.3%) | 0.56 | 11 (47.8%) | 33 (63.5%) | 0.009 | 7 (53.8%) | 37 (59.7%) | 0.19 |

**Table S2. Determinants of abnormal radiological signs in patients with definite non-occlusive mesenteric ischemia (n=75)**

The lactate level is expressed as median (interquartile range)





**Figure S1. Distribution of radiological signs in patients with definite non-occlusive mesenteric ischemia**

The Venn’s diagram was drawn from the 48 patients with definite non-occlusive mesenteric ischemia displaying at least one of the three most specific radiological signs.
